# Classical Swine Fever—An Updated Review

**DOI:** 10.3390/v9040086

**Published:** 2017-04-21

**Authors:** Sandra Blome, Christoph Staubach, Julia Henke, Jolene Carlson, Martin Beer

**Affiliations:** 1 Friedrich-Loeffler-Institut, Institute of Diagnostic Virology, Suedufer 10, 17493 Greifswald, Germany; julia.henke@fli.de (J.H.); jolene.carlson@fli.de (J.C.); martin.beer@fli.de (M.B.); 2 Friedrich-Loeffler-Institut, Institute of Epidemiology, Suedufer 10, 17493 Greifswald, Germany; christoph.staubach@fli.de

**Keywords:** porcine viruses, *Pestivirus*, classical swine fever, clinical signs, pathogenesis, epidemiology, diagnosis, control, vaccination, marker strategy

## Abstract

Classical swine fever (CSF) remains one of the most important transboundary viral diseases of swine worldwide. The causative agent is CSF virus, a small, enveloped RNA virus of the genus *Pestivirus*. Based on partial sequences, three genotypes can be distinguished that do not, however, directly correlate with virulence. Depending on both virus and host factors, a wide range of clinical syndromes can be observed and thus, laboratory confirmation is mandatory. To this means, both direct and indirect methods are utilized with an increasing degree of commercialization. Both infections in domestic pigs and wild boar are of great relevance; and wild boars are a reservoir host transmitting the virus sporadically also to pig farms. Control strategies for epidemic outbreaks in free countries are mainly based on classical intervention measures; i.e., quarantine and strict culling of affected herds. In these countries, vaccination is only an emergency option. However, live vaccines are used for controlling the disease in endemically infected regions in Asia, Eastern Europe, the Americas, and some African countries. Here, we will provide a concise, updated review on virus properties, clinical signs and pathology, epidemiology, pathogenesis and immune responses, diagnosis and vaccination possibilities.

## 1. Introduction

Classical swine fever (CSF) is one of the most important viral diseases of domestic pigs and wild boar. It has tremendous impact on animal health and pig industry and is therefore notifiable to the World Organization for Animal Health (OIE) [1]. After implementation of strict control measures, several countries succeeded in eradicating CSF. Nevertheless, in most parts of the world with significant pig production, CSF is at least sporadically present. Endemicity can be assumed in several countries of South and Central America, parts of Eastern Europe and neighboring countries, as well as Asia, including India. Little is known about the African situation.

A binding legal framework exists for the surveillance and control in most countries. Integral parts of the control measures are timely and reliable diagnosis, stamping out of infected herds, establishment of restriction zones, movement restrictions, and tracing of possible contacts. Prophylactic vaccination and other treatments are often also strictly prohibited. However, in Europe, where affected wild boar populations were shown to be an important reservoir for the virus, and acted as a source for reintroduction into the domestic pig population [2,3], emergency vaccination of wild boar has been practiced to control the disease [4,5,6,7]. Emergency vaccination is also among the options to combat CSF in domestic animals. Furthermore, vaccination is still in use to reduce the disease burden in endemically affected countries. 

Design of control measures and risk assessment depends on the knowledge of factors that influence disease dynamics and epidemiology. For this purpose, the presented review aims at providing an updated overview on the disease and the underlying mechanisms but also control and diagnostic options. 

## 2. Virus Properties

### 2.1. Virus Organization and Replication

*Classical swine fever virus* (CSFV) belongs to the genus *Pestivirus* within the *Flaviviridae* family [1]. Other members of this genus are *Bovine viral diarrhea virus* 1 and 2 (BVDV-1 and -2), *Border disease virus* (BDV) and a growing number of unclassified and so-called atypical pestiviruses, from giraffe-virus over HoBi-like viruses to recently discovered Bungowannah virus and atypical porcine pestivirus [2,3,4,5,6,7,8,9,10,11,12,13]. 

The enveloped viral particles consist of four structural proteins, namely the core protein (C), and envelope glycoproteins E1, E2, and E^rns^ [14,15,16,17,18]. The core encloses the positive single-stranded RNA genome of approximately 12.3 kb [19,20,21,22] which is translated into one polyprotein. The coding region is flanked by non-translated regions (NTR) at both ends. Co- and post-translational processing of the precursor protein by viral and cellular proteases results in 13 mature proteins, the above-mentioned structural proteins and non-structural proteins N^pro^, p7, NS2-3, NS2, NS3, NS4A, NS4B, NS5A, and NS5B. The latter have various functions in the viral replication, e.g., NS5B represents the RNA-dependent RNA polymerase [23] and NS3 acts as protease [24,25].

Virus replication takes place in the cytoplasm after receptor mediated endocytosis and does normally not lead to a cytopathic effect in cell culture (naturally occurring CSFV strains were found to be non-cytopathic) [26]. A putative receptor is the porcine complement regulatory protein cluster of differentiation (CD) 46 that was shown to play a major role in CSFV attachment, together with heparan sulfates [27]. Upon cell culture adaptation an increased usage of heparin sulfates is observed for cell-virus interaction [28]. The mutation responsible for the adaptation lies within the E^rns^ encoding region [8], namely in the C-terminus where a Ser residue is replaced by an Arg residue at amino acid 476 in the polyprotein of CSFV. 

In any case, glycoproteins E2 and E^rns^ are necessary for viral attachment [9,10], and the initial contact with the host cell is mediated through the E^rns^ which interacts with glycosaminoglycans [10,11]. For receptor binding and subsequent endocytosis, the E2-E1 heterodimer is essential [12]. After fusion of the virus envelope with the endosomal membrane, the virus core is released into the cytoplasm [13,14,15]. Thereafter, viral RNA is released into the cytoplasm and translation takes place. The binding of ribosomes at the rough endoplasmatic reticulum is realized through an internal ribosomal entry site (IRES) at the 5′ NTR, which allows a cap-independent translation [16,17,18]. The processing of the resulting viral polyprotein precursor occurs with the help of viral and cellular proteases [19]. Initially, autoproteinase N^pro^ is cleaved from the polyprotein [20,21]. Subsequently, cellular proteases cleave the C-protein and E^rns^, E1 and E2, E2 and p7 as well as NS2-3. NS2-3 is then partially processed through the autocatalytic cysteine protease activity of NS2 into NS2 and NS3. In this way NS2 generates its own C-terminal ending [22,23]. The serine protease activity of NS3 leads to the cleavage of the rest of the NS3-NS5 region [24]. While replication progresses, negative-stranded RNA is generated, which serves as template for the synthesis of the positive stranded RNA. The positive stranded RNA is then packed into the capsid [25]. Virion assembly and maturation takes place in the endoplasmatic reticulum and the Golgi apparatus after which the progeny virions bud at the cell membrane through exocytosis [26,27].

### 2.2. Tenacity and Virus Inactivation

The survival of CSFV under different ambient conditions varies considerably and is influenced especially by the temperature but also by the matrix in which it is found. Generally, survival times are higher under cold, moist and protein rich conditions [28]. The dependence of viral survival and temperature is well studied [29,30,31]. 

For animal disease control, survival in excretions (left in the pen or stored as slurry) and stability in meat products are crucial parameters. For CSFV in excretions, survival times were demonstrated that range from a few days at room temperature to several weeks at 5 °C [32]. If temperatures are higher than 35 °C, survival times are dramatically reduced, and inactivation occurs in hours or even minutes from temperatures above 50 °C [33]. This is an important factor when biogas plants and other industry parts are discussed. Along the same lines, Botner and Belsham [34] could show that survival of CSFV in slurry was short when heated and remained infective for weeks at cool temperature. Turner showed that complete inactivation was achieved at 60 °C for 3 min under lab conditions [35]. However, homogeneity of the mixture that is to be inactivated and thus temperature distribution is crucial [36]. For contaminated pig pens, this can mean virus survival for at least several days [37] to one month under cold winter conditions [38]. Under laboratory conditions, freeze-thawing has a negative impact on viral titers which can however be prevented by some chemical compounds such as dimethyl sulfoxide [39]. With regard to pH values, CSFV is relatively stable between pH 5 and 10. Half-lives at low pH levels are temperature dependent with mean half-lives that are more than ten-fold lower at room temperature compared to 4 °C (70 h at 4 °C compared to 5 h at 21 °C for pH 3). Overall variability is high and shows some strain dependence [40]. Another important matrix is meat or downstream products. Farez and Morley [30] report virus survival over years in meat frozen at −70 °C and of days to years in different meat products. Survival of 4.5 years in frozen meat was also reported by Edgar (reviewed in the EFSA scientific report 2009, [28]). Curing and smoking alone have little effect on the virus while higher temperatures readily inactivate the virus [31]. Survival times of more than 75 days were reported for salami [41] and over 120 days for Iberian loins or shoulders [42].

### 2.3. Genetic Diversity and Virulence Factors

Classical swine fever virus strains can be divided into three genotypes with three to four sub-genotypes. The most recently added sub-genotype 1.4 was only very recently described for CSFV strains from Cuba. These strains had so far been placed into sub-genotype 1.2 but formed a distinct cluster when compared based on longer genome fragments, e.g., full-length E2, N^pro^, C, E1, and E^rns^ [43]. Further divisions that have been proposed concern sub-genotypes 2.1 and 2.3 [44,45,46,47]. However, these systems of clusters or clades were not further harmonized and did not enter routine use. The genetic diversity does not result in true serotypes and does not impact vaccine efficacy. In general, CSFV is highly stable, especially for an RNA virus [48]. 

Up to very recently, phylogenetic studies were mainly based on two short fragments, namely a 150 nucleotide (nt) fragment of the 5′NTR and a 190 nt fragment of the E2 encoding region [49]. Moreover, a 409 nt fragment of the region coding for the polymerase gene NS5B was employed [50]. With the advent of affordable sequencing technologies for longer fragments or even full genomes, in-detail analyses are now more often based on more than the traditional fragments. The European Union (EU) Reference Laboratory for CSF nowadays recommends using full-length E2 encoding sequences for reliable CSFV phylogenies [51]. The latter resulted, e.g., in the designation of the above-mentioned new sub-genotype 1.4. Full-length sequences are being employed for quasispecies analyses, investigation of virulence determinants but also high resolution molecular epidemiology [52,53,54,55].

**Figure 1:**
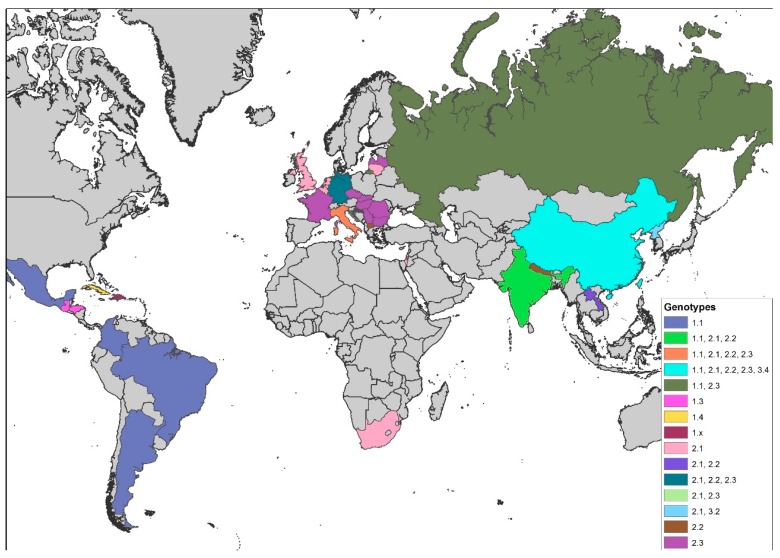
Global distribution of classical swine fever virus (CSFV) sub-genotypes (map based on Global Administrative Areas (GADM database 2.8; November 2015)).

The distribution of genotypes shows a distinct geographical pattern [50,56]: Whereas isolates belonging to group 3 seem to occur solely in Asia, all European CSFV isolates of the 1990s and later belonged to one of the subgroups within group 2 (2.1, 2.2, or 2.3) [45,51,57,58,59,60,61,62,63,64] and were clearly distinct from former CSF reference viruses, which belong to group 1 [50,65]. On the global scale, the most prevalent genotype over the last decades was undoubtedly genotype 2 [66]. However, all field isolates from the American continent belong to genotype 1 with only 1.1 strains from Argentina, Brazil, Colombia, and Mexico; 1.3 strains from Honduras and Guatemala; and the above-mentioned sub-genotype 1.4 strains from Cuba [43,67,68,69]. Little is known about the CSF situation in Africa and the Middle East. Exceptions are the 2005 outbreak in South Africa and the 2009 outbreak in Israel that were both caused by 2.1 CSFV strains [70,71]. Reports from India are increasingly detailed and demonstrate that sub-genotypes 1.1, 2.1, and 2.2 are co-circulating [72,73,74,75,76,77,78,79]. This changes the historical situation where genotype 1.1 strains predominated. From Nepal, strains of sub-genotype 2.2 were reported [80]. The situation in China is characterized by high variability of strains that belong mainly to sub-genotypes 1.1, 2.1, 2.2, and 2.3 [81,82,83,84]. Taiwan is also experiencing a change in sub-genotypes. It seems that the historical 3.4 strains are replaced by the Chinese 2.1 strains [85]. However, Taiwanese reports include all the above-mentioned sub-genotypes [85,86,87]. Sub-genotype 2.1 and 2.2 strains are also reported from Laos [88,89]. From Korea, strains of sub-genotypes 3.2 and 2.1 were reported [44], and, for Japan, indications exist that genotype 3 is found [90]. Generally, endemicity is accompanied or driven by strains of moderate or low virulence. These strains have been found in several regions with long-term circulation of CSFV (e.g., Cuba [91]), and mathematical models have shown that these strains may represent the viral optimum for long-term persistence [92]. An overview of the genotype distribution is provided in Figure 1.

European CSFV sequences were collected and made available through the semi-public CSF-database (DB) at the EU and OIE reference laboratory for CSF in Hannover, Germany [49,93,94,95]. Since the Institute of Virology became European Reference Laboratory for CSF more than 30 years ago, the virus isolates involved in European outbreaks but also other accessible sequence data were collected and stored. The database includes the above-mentioned fragments and also partial NS5B, full E2, and full-length CSFV sequences. It also allows automated typing and retrieval of sequences for in-detail analyses [95].

The search for virulence markers indicated a role of the N^pro^ [96], the E2 [97], the ribonuclease activity and dimerization of the E^rns^ [98,99], and NS4B [100]. Furthermore, glycosylation of structural proteins was shown to affect virulence [101,102,103,104,105]. However, these determinants are still far from being understood and do not seem to be transferrable among strains. Even the direct comparison of vaccine strains and their virulent ancestors did not reveal clear pattern [100,106]. Investigations into the role of quasispecies composition did not lead to the establishment of a clear correlation between variability and virulence [52]. There were also no predictors for different disease courses found [107].

## 3. Clinical Signs and Pathomorphological Lesions 

**Figure 2:**
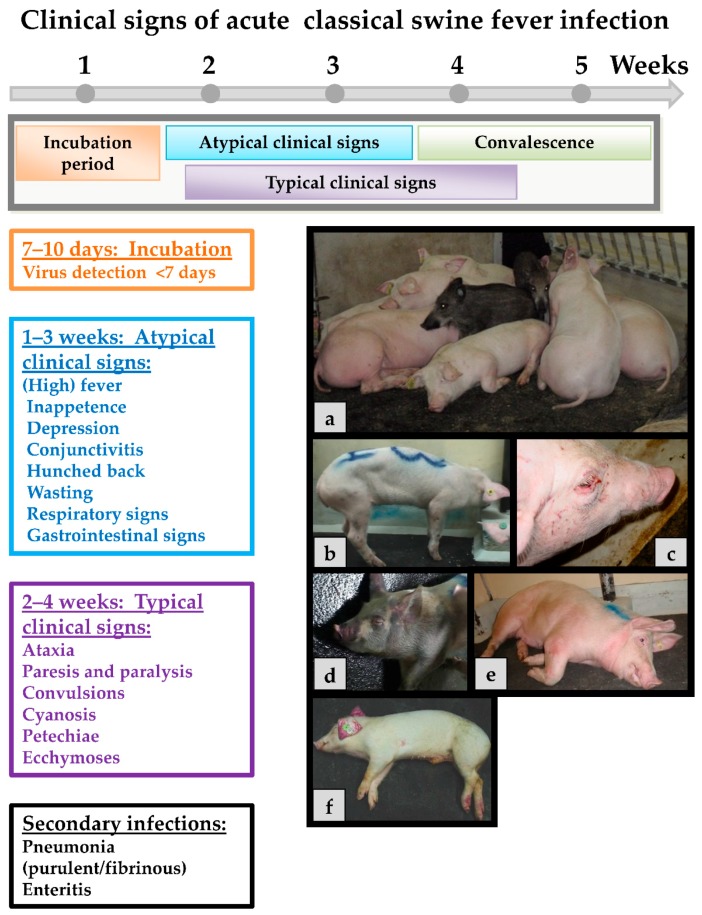
Acute CSFV infection with moderately virulent strains. The incubation period in most cases is from 7 to 10 days. Atypical clinical signs range from one to two weeks. Typical clinical signs occur around 2 to 4 weeks. The convalescent period is from 3 to 4 weeks. Death can occur as late as five weeks post-infection. (**a**) Swine are huddling, 10–15 days post-infection; (**b**) swine are presenting with hunched back; (**c**) severe conjunctivitis; (**d**) severe cyanosis of the skin around the face, ears, and limbs; (**e**) neurological signs, swine was unable to stand; and (**f**) dead swine with classic cyanosis of the ears.

Classical swine fever can be divided into the following forms of the disease: an acute (transient or lethal), a chronic and a persistent course, which usually requires infection during pregnancy [65]. In general, the same clinical signs are seen in both domestic pigs and wild boar, and show up after an incubation period of four to seven (seldom 10) days after the infection. The progression is dependent on strain virulence, host responses, and secondary infections and may vary considerably. However, infection of young pigs (weaners) with a moderately virulent CSFV strain may serve as an example for the acute disease course: During the first two weeks upon infection, the acute phase is characterized by unspecific (often referred to as “atypical”) clinical signs like high fever, anorexia, gastrointestinal symptoms, general weakness, and conjunctivitis [108]. Around two to four weeks after infection neurological signs can occur including incoordination, paresis, paralysis and convulsions. At the same time, skin hemorrhages or cyanosis can appear in different locations of the body such as the ears, limbs, and ventral abdomen. These late signs are the textbook cases and are therefore referred to as “typical” CSF signs. Examples of clinical signs can be found in Figure 2.

In acute-lethal courses, death usually occurs 2–4 weeks after CSFV infection. Mortality can reach up to 100% from 10 to 30 days depending on the age of the animal and the virulence of the virus strain [65,109,110,111]. Due to the immunosuppressive character of CSF infection, severe respiratory and gastrointestinal secondary infections can complicate the disease course and overlay the CSF signs. This is particularly important for clinical diagnosis. Infections with highly virulent CSFV strains such as “Margarita” or “Koslov” (the ones that are often used for vaccine testing) show a less age-dependent clinical course and may result in 100% mortality in all age classes of animals and severe neurological signs within 7 to 10 days (see, e.g., [112]).

**Figure 3:**
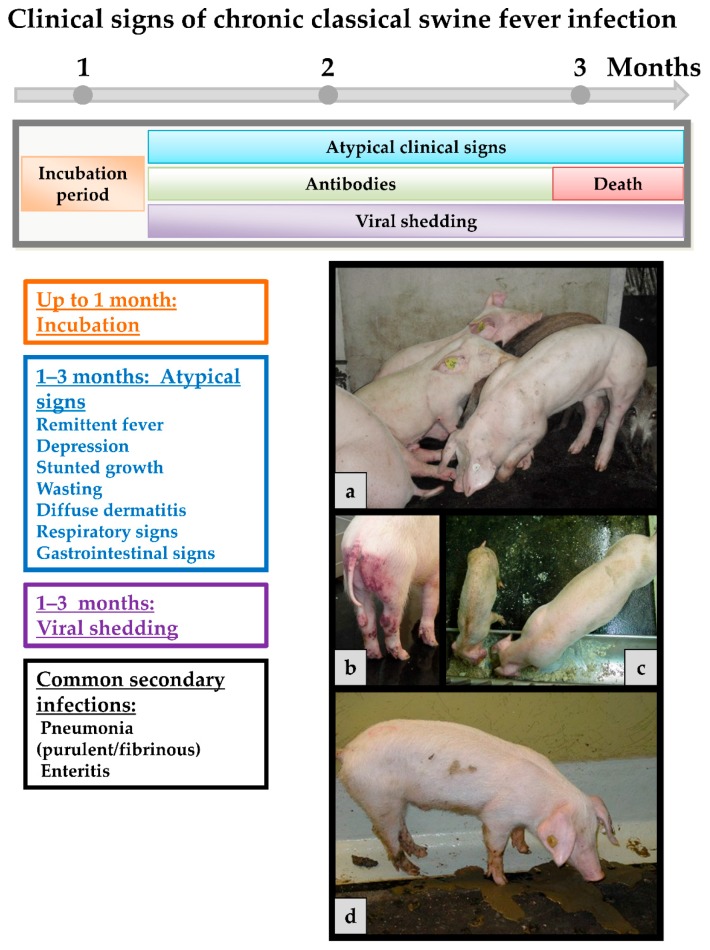
Chronic CSFV infection. The incubation period is the same as with the acute course. However, it may take up to a month until they are truly recognized. Atypical clinical signs can be present throughout and until death, occurring up to three months or even later after the infection. Antibodies can be detected at low levels after two weeks or later but do usually not persist. Viral shedding is observed from about four days post infection till the death of the animal. (**a**) Pigs are depressed, hunched over, and anorexic; (**b**) pig with petechial bleedings and ecchymosis in the anogenital region; (**c**) stunted and wasting pig beside a normally developed one of the same age; and (**d**) pig with diarrhea, shedding high viral loads until death.

Chronic course occurs when an infected pig is not able to mount an adequate immune response. In general, only non-specific clinical signs are observed in infected animals like remittent fever, depression, wasting and diffuse dermatitis (see Figure 3). It is acknowledged opinion that all chronically infected animals will eventually die. However, they can live for month in which they constantly shed high amounts of virus. Affected animals may develop antibodies that are in some cases only intermittently present and do not effect viral clearance. This, together with persistent infection, can play a role especially for affected wild boar populations [113,114,115], but also in endemically affected regions with constant virus circulation. Host rather than viral factors seem to play a role for the establishment of chronic infection [107]. 

When infection occurs during pregnancy, the virus can also infect the fetus in the womb due to its ability to pass the placental barrier which in turn might lead to persistent infection in the piglets. While the sows often show only mild clinical signs, an infection depending on the stage of gestation, leads to absorption or mummification of the fetuses and to abortions or stillbirth [114,116,117,118,119,120,121,122,123]. When infected between days 50 and 70 of pregnancy, an immunotolerance phenomenon can be induced and persistently infected offspring are born. The problem is that those piglets seem to be healthy and survive for several months but die due to the so-called late onset form of CSF. During that period they shed high viral loads which are sufficient for transmission. Recent studies discuss that persisting infection can also be induced when infecting newborn piglets within the first eight hours of life or even 48 h after birth [124,125]. This was shown to impact on the efficacy of vaccines and may complicate control in endemically affected countries.

**Figure 4:**
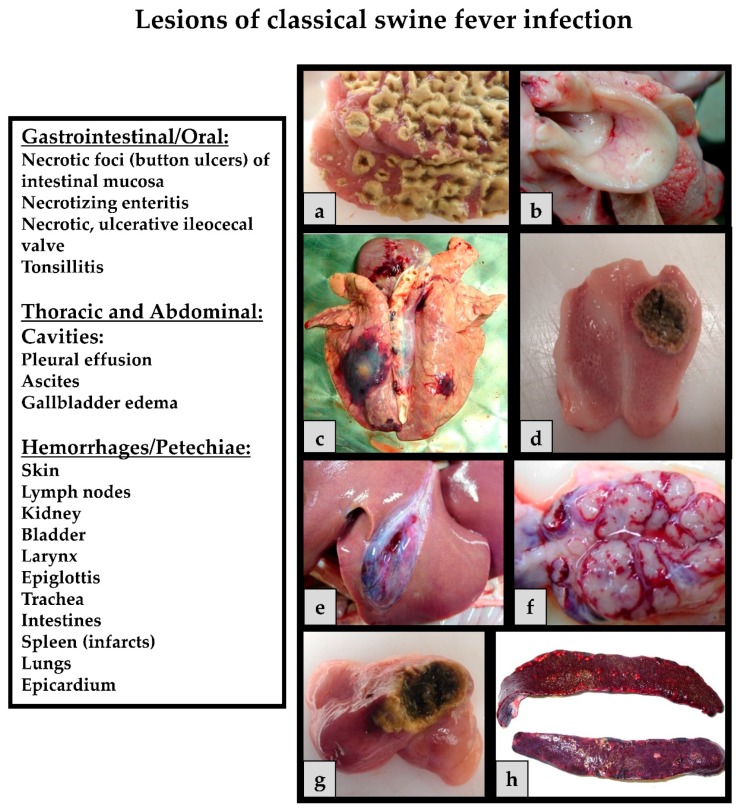
CSF related lesions: (**a**) Diphtheroid-necrotizing enteritis; (**b**) hemorrhages on the epiglottis; (**c**) severe secondary infections of the lung (*Actinobacillus pleuropneumoniae*); (**d**) necrotic tonsillitis with an ulcer; (**e**) gallbladder edema; (**f**) hemorrhagic lymph node; (**g**) necrotizing ileocecal valve; and (**h**) splenic infarcts.

The pathological findings (Figure 4) depend on the course of the viral infection. In the acute course of CSF, pathology often reveals enlarged lymph nodes, hemorrhages and petechiae on serosal and mucosal surfaces of different organs such as the, lungs, kidneys, intestines and urinary bladder. Tonsillitis, necrotic ulcers in the intestines, lesions in the lymphoreticular system, and non-purulent encephalitis can be observed [126] Splenic infarctions can occur and are considered pathognomic for CSF [127]. Infected piglets develop leukopenia, thrombocytopenia and immunosuppression, which increases the risk for secondary infections and thus to diseases of the gastrointestinal and respiratory system [128]. In the chronic form, pathological lesions include atrophy of the thymus, depletion of the lymphoid organs, necrosis and ulceration of the small intestine, colon, and ileocecal valve. It is important to consider that these clinical signs and pathological lesions should be considered as differentials for a number of swine pathogens. These unspecific clinical signs and lesions can vary among animals depending on host factors and the virulence of the CSFV strain. Often, the age, breed and immune status play a role in the outcome of the disease [65,108,129].

## 4. Pathogenesis and Immune Responses

As mentioned above, clinical signs of CSFV infections can vary considerably from peracute deaths to unapparent courses depending on virulence of the virus strain involved and different (partly unknown) host factors [65]. Unspecific clinical signs predominate, and differentiation from several other infectious diseases of swine is only possibly based on laboratory diagnosis. Acute-lethal forms can be viral hemorrhagic fever-like with severe thrombocytopenia, pulmonary edema, petechial bleedings, and increased vascular leakage [130]. Cytokine involvement is discussed for many lesions observed in acute CSF [131].

Infection with CSFV is followed by primary replication in the tonsils and subsequently spread to surrounding lymphoid tissues [132]. The virus reaches the regional lymph nodes through lymphatic vessels. Here further replication takes place and the virus is spread via blood to secondary replication sites such as spleen, bone marrow, and visceral lymph nodes [133,134,135]. Apoptotic reactions as well as phagocytic and secretory activation can be observed in several macrophage populations [136,137,138,139,140,141,142,143,144]. These activated macrophages seem to play a crucial role in (immuno-)pathogenesis while direct damage by the virus could be almost excluded for many lesions occurring in the course of CSFV infection. Moreover, dendritic cells are targeted and disturbance of the interferon system contributes to the pathogenesis [136,137,138,139,140]. There seems to be a correlation between high interferon (IFN)-α in the serum and disease severity and virulence of the strain involved [140,141]. High IFN-α concentrations are found as early as two days post infection, prior to the onset of clinical symptoms [112]. These findings are confirmed by microarray analyses of peripheral blood monocytic cells derived from CSFV-infected pigs [142].

Especially in the acute-lethal course, CSF is accompanied by severe lymphopenia and resulting immunosuppression as well as granulocytopenia [143,144,145,146]. Moreover, a marked thrombocytopenia starts very early after infection [147,148,149]. The mechanisms leading to this platelet decrease are not yet understood but disseminated intravascular coagulation (DIC), degeneration of megakaryocytes, bone marrow lesions, and accelerated deterioration have been discussed [130]. In addition, massive activation and subsequent phagocytosis of platelets has been discussed as an etiological factor [147] while DIC related correlates were not observed upon infection with a genotype 2.3 CSFV strain [150]. At least in vitro, endothelial cells are also activated and expression levels of pro-inflammatory and pro-coagulatory factors are increased [151]. The pathogenic mechanism involved in hemorrhagic lesions include damage of endothelial cells, causal involvement of thrombocytopenia (and DIC), erythrodiapedesis, and capillary vasodilatation and increased permeability [146,148,149,152,153]. However, several factors remain unclear and studies with different strains have given conflicting results.

Despite the immunopathogenesis of most CSF-related lesions, pigs recovering from CSFV infection mount an effective immune response with E2-specific antibodies detectable after 10–14 days. The E2 antibodies are able to neutralize CSFV in vitro and induce protective immune responses [154,155]. These antibodies and protection against re-infection persist probably livelong. In addition to E2, antibodies are raised against the E^rns^ and the non-structural protein NS3 [156,157]. Immunization with live attenuated CSFV can be efficient as early as 3–5 days post vaccination [158,159,160]. Thus, protection is possible without neutralizing antibodies and even before specific T-cell responses can be seen. Despite the fact that this very early protection is far from being understood, IFN-γ secreting T-cells seem to play a role [161,162,163]. 

## 5. Epidemiology

Susceptible hosts are different members of the *Suidae* family, particularly domestic pigs (*Sus scrofa domesticus*) and European wild boar (*Sus scrofa scrofa*) [113,164]. Moreover, the susceptibility of common warthogs (*Phacochoerus africanus*) and bushpigs (*Potamochoerus larvatus*) was recently demonstrated [165].

Classical swine fever virus can be transmitted both horizontally and vertically. Horizontal transmission takes places through direct or indirect contact between infected and susceptible pigs. Important indirect routes include feeding of virus contaminated garbage/swill and mechanical transmission via contact to humans or agricultural and veterinary equipment [127]. Aerogenic transmission was reported under experimental conditions [166,167,168], and it can probably play a role for within herd transmission [169]. 

Upon contact, infection usually occurs through the oronasal route, or less frequently via conjunctiva, mucus membranes, skin abrasions, insemination, and the use of contaminated instruments [170,171,172,173]. Infected pigs show high-titer viremia and shed virus at least from the beginning of clinical disease until death or specific antibodies have developed. The main excretion routes are by saliva, lacrimal secretions, urine, feces, and semen [127,135,173]. As mentioned above, chronically infected pigs shed the virus continuously or intermittently until death [65]. Vertical transmission from pregnant sows to fetuses is possible throughout all stages of gestation and can lead to persistently infected offspring (see above).

Classical swine fever affected wild boar populations can serve as reservoir of the virus and present a constant risk for domestic pigs. Fritzemeier et al. [2] could show that almost 60% of the primary CSF outbreaks in Germany between 1993 and 1998 were linked to infected wild boar. This link was particularly important for holdings with low biosecurity or problems in biosafety management. 

Over the last decades, a decreasing virulence was observed for the CSFV strains involved in many outbreaks among wild boar and domestic pigs. In Europe, the most prevalent genotype 2.3 strains showed moderate virulence with a highly age-dependent clinical picture and rather unspecific clinical pictures in older animals (see above). These strains showed potential to establish endemicity in affected wild boar populations rather than showing the self-limiting behavior of the historical highly virulent CSFV strains. It was discussed whether these strains are somewhat the ideally adapted variants of CSFV for long-term perpetuation in wildlife [92]. 

In endemically affected countries with official but imperfect vaccination, circulation of less virulent CSFV strains is often masked by partial protection. In combination with management and biosecurity issues (swill feeding, contacts, shared equipment), the virus is maintained over prolonged periods in the domestic pig population. 

## 6. Diagnosis

Rapid and reliable diagnosis is of utmost importance for the timely implementation of control measures against CSF. On the international level, laboratory methods as well as sampling and shipping guidelines can be found in the OIE Manual of Diagnostic Tests and Vaccines for Terrestrial Animals and the respective EU Diagnostic Manual (European Commission Decision 2002/106/EC). 

For CSFV, primary detection is performed using well established real-time reverse transcription polymerase chain reaction (RT-qPCR) systems [174,175,176,177,178,179,180,181,182,183], of which many are available commercially. Recently, field applicable RT-PCRs [184] but also alternatives have been designed such as loop-mediated isothermal amplification (LAMP) assays [185,186,187,188,189,190], primer-probe energy transfer RT-qPCR [191,192] or recently insulated isothermal RT-qPCR [193]. Moreover, CSFV can be isolated on different permanent cell lines such as porcine kidney cell lines PK15 or SK6 (Technical Annex to Commission Decision 2002/106/EC). In addition, detection of antigen on fixed cryosections of tissues is possible using fluorescence antibody or immune-peroxidase assays [194,195]. The available antigen ELISAs are recommended for the use with herd-based testing only. While the sensitivity of panpesti-specific assays (based on the E^rns^) is usually at least comparable with virus isolation, most CSF specific assays lack sensitivity [196]. Serological screening can be performed using different commercially available E2 antibody enzyme-linked immunosorbent assays (ELISAs). In addition, neutralization assays allow, to a certain extent, differentiation of pestivirus antibodies and are used for confirmation [197].

Reliable DIVA (differentiation of infected and vaccinated animals) assays are needed when using DIVA vaccines. Commercially available tests that can accompany both E2 subunit vaccines and chimeric vaccines such as “CP7_E2alf”, target the detection of antibodies directed against glycoprotein E^rns^ [196,198,199]. Recently, additional diagnostic tests have been developed. One is a double-antigen ELISA format that was recently commercialized [200], another is an ELISA with a screening and a confirmation part [201]: Moreover, a microsphere immunoassay was also developed as a confirmatory test [202]. 

Due to the increased sensitivity of diagnostic tools (especially RT-qPCR), vaccine virus detections are quite common in oral vaccination campaigns of wild boar and vaccination programs of domestic pigs. For this reason, different RT-qPCR systems have been developed and tested, these allow differentiation between vaccine and field viruses (genetic DIVA) [203,204,205,206,207,208].

Sampling can be the bottleneck of swine fever diagnosis, especially in the case of wild boar, but also in remote areas. For this reason, alternative sampling strategies and sample matrices have been tested for CSF (often combined with African swine fever sampling) especially for wildlife specimens and under rural conditions [209,210,211,212]. However, most of them are not yet in routine use and need further validation.

## 7. Vaccination

Highly efficacious and safe live-attenuated CSF vaccines have existed for decades [160]. The underlying virus strains (e.g., the C-strain of CSFV, the Lapinized Philippines Coronel, the Thiverval or the Japanese guinea-pig exaltation negative GPE strain) were attenuated through serial passages in animals (rabbits) or cell culture. These vaccines have been implemented in mandatory control programs that led, together with strict hygiene measures, to the eradication of CSF from several regions of the world [213]. At this time, they are still in use in several Asian countries including China [84], countries of South and Central America, Trans-Caucasian Countries, and Eastern Europe (see Table 1). In 2016, 22 countries officially reported mandatory vaccination campaigns (OIE WAHIS [214]). 

In addition, these vaccines were also adapted to a bait format for oral immunization of wild boar [6,215,216] and were recently explored for the vaccination of domestic pigs under backyard conditions [217,218,219]. While these vaccines usually have outstanding virtues in terms of onset, spectrum and duration of immunity [158,220,221,222,223], the main drawback is the lack of a serological marker concept [160] that would allow differentiation of field virus infected from vaccinated animals (DIVA concept). This is usually less important in endemically affected countries where prophylactic vaccination is carried out to reduce the disease burden and to ensure product safety. In general, there are also no legal obligations to use a certain type of vaccine for an emergency vaccination scenario. However, due to the trade restrictions that are imposed on pigs vaccinated with conventional live attenuated vaccines, only DIVA vaccines are considered a feasible option for domestic pigs [224]. Up to very recently, only E2 subunit marker (DIVA) vaccines were available on the market (at present, one E2 marker vaccine is commercially available, Porcilis^®^ Pesti, MSD Animal Health, Unterschleißheim, Germany). These vaccines are safe and were shown to provide clinical protection and limit the spread of CSF [225,226,227,228,229,230,231,232,233,234,235]. However, they show drawbacks especially in terms of early protection [160,236] and protection against transplacental transmission [237]. Due to these problems, emergency vaccination was hardly implemented in domestic pigs (one exception being Romania). Several research groups have therefore sought to develop a next-generation marker vaccine candidate that would ideally answer all demands with regard to safety, efficacy, DIVA potential, and marketability [238]. Among the concepts that have been investigated are different vector vaccines based on vaccinia virus, pseudorabies virus or adenoviruses. Other vaccine designs include recombinant attenuated vaccines with chimeric constructs, subunit vaccines based on different expression systems, and RNA/DNA vaccines (recently reviewed by Blome et al., [239]. In 2014, the European Medicines Agency (EMA) licensed one of the chimeric marker vaccine candidates, “CP7_E2alf”, after extensive testing in the framework of an EU-funded research project [159,240,241,242,243,244,245,246,247,248,249,250,251,252,253,254,255,256,257]. This new marker vaccine is still under investigation and could be a powerful tool for both emergency vaccination of domestic pigs and also wild boar. 

Oral emergency vaccination of wild boar with baits has proven to be a potent tool to control the disease in wildlife and to safeguard domestic pigs [3]. For this purpose, the above-mentioned C-strain formulations have been used in several European countries including Germany and France. To further optimize the strategy, a DIVA vaccine such as “CP7_E2alf” could be used. The latter was already tested for use in wild boar under both laboratory and field conditions and could be a medium term option [241,246,251]. 

## Figures and Tables

**Table 1:** CSF vaccination: Countries that reported official vaccination campaigns through World Organization for Animal Health (OIE) in 2016 (their last reported outbreaks are presented in brackets; no reports for some countries since 2005) (WAHIS Interface [214]).

Country	Last reported CSF outbreak
Albania	no reports
Armenia	2006
Azerbaijan	no reports
Belarus	no reports
Bosnia and Herzegovina	2007
Bulgaria (wb)	2009 wb
China	2015
Colombia	2016
Cuba	2016
Dominican Republic	2016
Ecuador	2016
Macedonia	2008
Georgia	no reports
Hong Kong	2005
Madagascar	2016
Moldova	(no reports)
Mongolia	2016
Myanmar	2015
Peru	2016
Philippines	2016
Russia	2016
Ukraine	2015

Wb: Wild boar.

## References

[1] Edwards S., Fukusho A., Lefevre P.C., Lipowski A., Pejsak Z., Roehe P., Westergaard J. (2000). Classical swine fever: The global situation. Vet. Microbiol..

[2] Fritzemeier J., Teuffert J., Greiser-Wilke I., Staubach C., Schlüter H., Moennig V. (2000). Epidemiology of classical swine fever in germany in the 1990s. Vet. Microbiol..

[3] Rossi S., Staubach C., Blome S., Guberti V., Thulke H.H., Vos A., Koenen F., Le Potier M.F. (2015). Controlling of csfv in european wild boar using oral vaccination: A review. Front. Microbiol..

[4] Rossi S., Pol F., Forot B., Masse-Provin N., Rigaux S., Bronner A., Le Potier M.F. (2010). Preventive vaccination contributes to control classical swine fever in wild boar (*Sus scrofa* sp.). Vet. Microbiol..

[5] von Rüden S., Staubach C., Kaden V., Hess R.G., Blicke J., Kühne S., Sonnenburg J., Fröhlich A., Teuffert J., Moennig V. (2008). Retrospective analysis of the oral immunisation of wild boar populations against classical swine fever virus (csfv) in region Eifel of Rhineland-Palatinate. Vet. Microbiol..

[6] Kaden V., Heyne H., Kiupel H., Letz W., Kern B., Lemmer U., Gossger K., Rothe A., Böhme H., Tyrpe P. (2002). Oral immunisation of wild boar against classical swine fever: Concluding analysis of the recent field trials in Germany. Berl. Munch. Tierarztl. Wochenschr..

[7] Blome S., Gabriel C., Staubach C., Leifer I., Strebelow G., Beer M. (2011). Genetic differentiation of infected from vaccinated animals after implementation of an emergency vaccination strategy against classical swine fever in wild boar. Vet. Microbiol..

[8] Hulst M.M., van Gennip H.G., Moormann R.J. (2000). Passage of classical swine fever virus in cultured swine kidney cells selects virus variants that bind to heparan sulfate due to a single amino acid change in envelope protein E^rns^. J. Virol..

[9] Weiland E., Ahl R., Stark R., Weiland F., Thiel H.J. (1992). A second envelope glycoprotein mediates neutralization of a pestivirus, hog cholera virus. J. Virol..

[10] Iqbal M., Flick-Smith H., McCauley J.W. (2000). Interactions of bovine viral diarrhoea virus glycoprotein E^rns^ with cell surface glycosaminoglycans. J. Gen. Virol..

[11] Hulst M.M., van Gennip H.G., Vlot A.C., Schooten E., de Smit A.J., Moormann R.J. (2001). Interaction of classical swine fever virus with membrane-associated heparan sulfate: Role for virus replication in vivo and virulence. J. Virol..

[12] Wang Z., Nie Y., Wang P., Ding M., Deng H. (2004). Characterization of classical swine fever virus entry by using pseudotyped viruses: E1 and E2 are sufficient to mediate viral entry. Virology.

[13] Donis R.O. (1995). Molecular biology of bovine viral diarrhea virus and its interactions with the host. Vet. Clin. N. Am. Food Anim. Pract..

[14] Krey T., Thiel H.J., Rümenapf T. (2005). Acid-resistant bovine pestivirus requires activation for ph-triggered fusion during entry. J. Virol..

[15] Lecot S., Belouzard S., Dubuisson J., Rouille Y. (2005). Bovine viral diarrhea virus entry is dependent on clathrin-mediated endocytosis. J. Virol..

[16] Rijnbrand R., van der Straaten T., van Rijn P.A., Spaan W.J., Bredenbeek P.J. (1997). Internal entry of ribosomes is directed by the 5′ noncoding region of classical swine fever virus and is dependent on the presence of an RNA pseudoknot upstream of the initiation codon. J. Virol..

[17] Pestova T.V., Hellen C.U. (1999). Internal initiation of translation of bovine viral diarrhea virus RNA. Virology.

[18] Poole T.L., Wang C., Popp R.A., Potgieter L.N., Siddiqui A., Collett M.S. (1995). Pestivirus translation initiation occurs by internal ribosome entry. Virology.

[19] Rümenapf T., Unger G., Strauss J.H., Thiel H.J. (1993). Processing of the envelope glycoproteins of pestiviruses. J. Virol..

[20] Stark R., Meyers G., Rümenapf T., Thiel H.J. (1993). Processing of pestivirus polyprotein: Cleavage site between autoprotease and nucleocapsid protein of classical swine fever virus. J. Virol..

[21] Wiskerchen M., Belzer S.K., Collett M.S. (1991). Pestivirus gene expression: The first protein product of the bovine viral diarrhea virus large open reading frame, p20, possesses proteolytic activity. J. Virol..

[22] Lackner T., Müller A., Pankraz A., Becher P., Thiel H.J., Gorbalenya A.E., Tautz N. (2004). Temporal modulation of an autoprotease is crucial for replication and pathogenicity of an rna virus. J. Virol..

[23] Lackner T., Thiel H.J., Tautz N. (2006). Dissection of a viral autoprotease elucidates a function of a cellular chaperone in proteolysis. Proc. Natl. Acad. Sci. USA.

[24] Tautz N., Elbers K., Stoll D., Meyers G., Thiel H.J. (1997). Serine protease of pestiviruses: Determination of cleavage sites. J. Virol..

[25] Gong Y., Trowbridge R., Macnaughton T.B., Westaway E.G., Shannon A.D., Gowans E.J. (1996). Characterization of RNA synthesis during a one-step growth curve and of the replication mechanism of bovine viral diarrhoea virus. J. Gen. Virol..

[26] Gray E.W., Nettleton P.F. (1987). The ultrastructure of cell cultures infected with border disease and bovine virus diarrhoea viruses. J. Gen. Virol..

[27] Ohmann H.B. (1990). Electron microscopy of bovine virus diarrhoea virus. Rev. Sci. Tech..

[28] Kramer M., Staubach C., Koenen F., Haegeman A., Pol F., Le Potier M.F., Greiser-Wilke I. (2009). Scientific review on Classical Swine Fever. EFSA Support. Publ..

[29] Wijnker J.J., Depner K.R., Berends B.R. (2008). Inactivation of classical swine fever virus in porcine casing preserved in salt. Int. J. Food Microbiol..

[30] Farez S., Morley R.S. (1997). Potential animal health hazards of pork and pork products. Rev. Sci. Tech..

[31] Edwards S. (2000). Survival and inactivation of classical swine fever virus. Vet. Microbiol..

[32] Weesendorp E., Stegeman A., Loeffen W.L. (2008). Survival of classical swine fever virus at various temperatures in faeces and urine derived from experimentally infected pigs. Vet. Microbiol..

[33] Haas B., Ahl R., Böhm R., Strauch D. (1995). Inactivation of viruses in liquid manure. Rev. Sci. Tech..

[34] Botner A., Belsham G.J. (2012). Virus survival in slurry: Analysis of the stability of foot-and-mouth disease, classical swine fever, bovine viral diarrhoea and swine influenza viruses. Vet. Microbiol..

[35] Turner C., Williams S.M., Cumby T.R. (2000). The inactivation of foot and mouth disease, aujeszky's disease and classical swine fever viruses in pig slurry. J. Appl. Microbiol..

[36] Gale P. (2004). Risks to farm animals from pathogens in composted catering waste containing meat. Vet. Rec..

[37] Artois M., Depner K.R., Guberti V., Hars J., Rossi S., Rutili D. (2002). Classical swine fever (hog cholera) in wild boar in Europe. Rev. Sci. Tech..

[38] Harkness J.W. (1985). Classical swine fever and its diagnosis: A current view. Vet. Rec..

[39] Tessler J., Stewart W.C., Kresse J.I. (1975). Stabilization of hog cholera virus by dimethyl sulfoxide. Can. J. Comp. Med..

[40] Depner K., Bauer T., Liess B. (1992). Thermal and pH stability of pestiviruses. Rev. Sci. Tech..

[41] Panina G.F., Civardi A., Cordioli P., Massirio I., Scatozza F., Baldini P., Palmia F. (1992). Survival of hog cholera virus (HCV) in sausage meat products (italian salami). Int. J. Food Microbiol..

[42] Mebus C., House C., Gonzalvo F.R., Pineda J., Tapiador J., Pire J., Bergada J., Yedloutschnig R., Sahu S., Becerra V. (1993). Survival of foot-and-mouth disease, african swine fever, and hog cholera viruses in spanish serrano cured hams and Iberian cured hams, shoulders and loins. Food Microbiol..

[43] Postel A., Schmeiser S., Perera C.L., Rodriguez L.J., Frias-Lepoureau M.T., Becher P. (2013). Classical swine fever virus isolates from Cuba form a new subgenotype 1.4. Vet. Microbiol..

[44] Cha S.H., Choi E.J., Park J.H., Yoon S.R., Kwon J.H., Yoon K.J., Song J.Y. (2007). Phylogenetic characterization of classical swine fever viruses isolated in Korea between 1988 and 2003. Virus Res..

[45] Blome S., Grotha I., Moennig V., Greiser-Wilke I. (2010). Classical swine fever virus in South-Eastern Europe—Retrospective analysis of the disease situation and molecular epidemiology. Vet. Microbiol..

[46] Chen N., Hu H., Zhang Z., Shuai J., Jiang L., Fang W. (2008). Genetic diversity of the envelope glycoprotein E2 of classical swine fever virus: Recent isolates branched away from historical and vaccine strains. Vet. Microbiol..

[47] Jiang D.L., Liu G.H., Gong W.J., Li R.C., Hu Y.F., Tu C., Yu X.L. (2013). Complete genome sequences of classical swine fever virus isolates belonging to a new subgenotype, 2.1c, from Hunan province, China. Genome Announc..

[48] Vanderhallen H., Mittelholzer C., Hofmann M.A., Koenen F. (1999). Classical swine fever virus is genetically stable in vitro and in vivo. Arch. Virol..

[49] Greiser-Wilke I., Dreier S., Haas L., Zimmermann B. (2006). Genetic typing of classical swine fever viruses—A review. Dtsch. Tierarztl. Wochenschr..

[50] Paton D.J., McGoldrick A., Greiser-Wilke I., Parchariyanon S., Song J.Y., Liou P.P., Stadejek T., Lowings J.P., Bjorklund H., Belak S. (2000). Genetic typing of classical swine fever virus. Vet. Microbiol..

[51] Postel A., Schmeiser S., Bernau J., Meindl-Boehmer A., Pridotkas G., Dirbakova Z., Mojzis M., Becher P. (2012). Improved strategy for phylogenetic analysis of classical swine fever virus based on full-length E2 encoding sequences. Vet. Res..

[52] Töpfer A., Höper D., Blome S., Beer M., Beerenwinkel N., Ruggli N., Leifer I. (2013). Sequencing approach to analyze the role of quasispecies for classical swine fever. Virology.

[53] Leifer I., Hoeper D., Blome S., Beer M., Ruggli N. (2011). Clustering of classical swine fever virus isolates by codon pair bias. BMC Res. Notes.

[54] Goller K.V., Gabriel C., Dimna M.L., Potier M.F., Rossi S., Staubach C., Merboth M., Beer M., Blome S. (2016). Evolution and molecular epidemiology of classical swine fever virus during a multi-annual outbreak amongst european wild boar. J. Gen. Virol..

[55] Fahnoe U., Pedersen A.G., Risager P.C., Nielsen J., Belsham G.J., Höper D., Beer M., Rasmussen T.B. (2014). Rescue of the highly virulent classical swine fever virus strain "Koslov" from cloned cDNA and first insights into genome variations relevant for virulence. Virology.

[56] Björklund H., Lowings P., Stadejek T., Vilcek S., Greiser-Wilke I., Paton D., Belak S. (1999). Phylogenetic comparison and molecular epidemiology of classical swine fever virus. Virus Genes.

[57] Greiser-Wilke I., Fritzemeier J., Koenen F., Vanderhallen H., Rutili D., De Mia G.M., Romero L., Rosell R., Sanchez-Vizcaino J.M., San Gabriel A. (2000). Molecular epidemiology of a large classical swine fever epidemic in the European Union in 1997–1998. Vet. Microbiol..

[58] Jemersic L., Greiser-Wilke I., Barlic-Maganja D., Lojkic M., Madic J., Terzic S., Grom J. (2003). Genetic typing of recent classical swine fever virus isolates from Croatia. Vet. Microbiol..

[59] Bartak P., Greiser-Wilke I. (2000). Genetic typing of classical swine fever virus isolates from the territory of the Czech Republic. Vet. Microbiol..

[60] Wonnemann H., Floegel-Niesmann G., Moennig V., Greiser-Wilke I. (2001). Genetic typing of German isolates of classical swine fever virus. Dtsch. Tierarztl. Wochenschr..

[61] Biagetti M., Greiser-Wilke I., Rutili D. (2001). Molecular epidemiology of classical swine fever in Italy. Vet. Microbiol..

[62] Pol F., Rossi S., Mesplede A., Kuntz-Simon G., Le Potier M.F. (2008). Two outbreaks of classical swine fever in wild boar in France. Vet. Rec..

[63] Leifer I., Hoffmann B., Höper D., Bruun Rasmussen T., Blome S., Strebelow G., Höreth-Böntgen D., Staubach C., Beer M. (2010). Molecular epidemiology of current classical swine fever virus isolates of wild boar in germany. J. Gen. Virol..

[64] Simon G., Le Dimna M., Le Potier M.F., Pol F. (2013). Molecular tracing of classical swine fever viruses isolated from wild boars and pigs in France from 2002 to 2011. Vet. Microbiol..

[65] Moennig V., Floegel-Niesmann G., Greiser-Wilke I. (2003). Clinical signs and epidemiology of classical swine fever: A review of new knowledge. Vet. J..

[66] Beer M., Goller K.V., Staubach C., Blome S. (2015). Genetic variability and distribution of classical swine fever virus. Anim. Health Res. Rev..

[67] Pereda A.J., Greiser-Wilke I., Schmitt B., Rincon M.A., Mogollon J.D., Sabogal Z.Y., Lora A.M., Sanguinetti H., Piccone M.E. (2005). Phylogenetic analysis of classical swine fever virus (CSFV) field isolates from outbreaks in south and central America. Virus Res..

[68] Diaz de Arce H., Nunez J.I., Ganges L., Barreras M., Teresa Frias M., Sobrino F. (1999). Molecular epidemiology of classical swine fever in Cuba. Virus Res..

[69] de Arce H.D., Ganges L., Barrera M., Naranjo D., Sobrino F., Frias M.T., Nunez J.I. (2005). Origin and evolution of viruses causing classical swine fever in Cuba. Virus Res..

[70] Sandvik T., Crooke H., Drew T.W., Blome S., Greiser-Wilke I., Moennig V., Gous T.A., Gers S., Kitching J.A., Buhrmann G. (2005). Classical swine fever in South Africa after 87 years' absence. Vet. Rec..

[71] David D., Edri N., Yakobson B.A., Bombarov V., King R., Davidson I., Pozzi P., Hadani Y., Bellaiche M., Schmeiser S. (2011). Emergence of classical swine fever virus in Israel in 2009. Vet. J..

[72] Barman N.N., Bora D.P., Khatoon E., Mandal S., Rakshit A., Rajbongshi G., Depner K., Chakraborty A., Kumar S. (2014). Classical swine fever in wild hog: Report of its prevalence in northeast India. Transbound. Emerg. Dis..

[73] Roychoudhury P., Sarma D.K., Rajkhowa S., Munir M., Kuchipudi S.V. (2014). Predominance of genotype 1.1 and emergence of genotype 2.2 classical swine fever viruses in north-eastern region of India. Transbound. Emerg. Dis..

[74] Patil S.S., Hemadri D., Shankar B.P., Raghavendra A.G., Veeresh H., Sindhoora B., Chandan S., Sreekala K., Gajendragad M.R., Prabhudas K. (2010). Genetic typing of recent classical swine fever isolates from India. Vet. Microbiol..

[75] Patil S.S., Hemadri D., Veeresh H., Sreekala K., Gajendragad M.R., Prabhudas K. (2012). Phylogenetic analysis of NS5B gene of classical swine fever virus isolates indicates plausible Chinese origin of Indian subgroup 2.2 viruses. Virus Genes.

[76] Rajkhowa T.K., Hauhnar L., Lalrohlua I., Mohanarao G.J. (2014). Emergence of 2.1. Subgenotype of classical swine fever virus in pig population of India in 2011. Vet. Q..

[77] Desai G.S., Sharma A., Kataria R.S., Barman N.N., Tiwari A.K. (2010). 5′ UTR-based phylogenetic analysis of classical swine fever virus isolates from India. Acta Virol..

[78] Sarma D.K., Mishra N., Vilcek S., Rajukumar K., Behera S.P., Nema R.K., Dubey P., Dubey S.C. (2011). Phylogenetic analysis of recent classical swine fever virus (CSFV) isolates from Assam, India. Comp. Immunol. Microbiol. Infect. Dis..

[79] Nandi S., Muthuchelvan D., Ahuja A., Bisht S., Chander V., Pandey A.B., Singh R.K. (2011). Prevalence of classical swine fever virus in India: A 6-year study (2004–2010). Transbound. Emerg. Dis..

[80] Postel A., Jha V.C., Schmeiser S., Becher P. (2013). First molecular identification and characterization of classical swine fever virus isolates from Nepal. Arch. Virol..

[81] An T.Q., Peng J.M., Tian Z.J., Zhao H.Y., Li N., Liu Y.M., Chen J.Z., Leng C.L., Sun Y., Chang D. (2013). Pseudorabies virus variant in Bartha-K61-vaccinated pigs, China, 2012. Emerg. Infect. Dis..

[82] Afshar A., Dulac G.C., Dubuc C., Howard T.H. (1991). Comparative evaluation of the fluorescent antibody test and microtiter immunoperoxidase assay for detection of bovine viral diarrhea virus from bull semen. Can. J. Vet. Res..

[83] Luo T.R., Liao S.H., Wu X.S., Feng L., Yuan Z.X., Li H., Liang J.J., Meng X.M., Zhang H.Y. (2011). Phylogenetic analysis of the *E2* gene of classical swine fever virus from the Guangxi province of southern China. Virus Genes.

[84] Luo Y., Li S., Sun Y., Qiu H.J. (2014). Classical swine fever in China: A minireview. Vet. Microbiol..

[85] Pan C.H., Jong M.H., Huang T.S., Liu H.F., Lin S.Y., Lai S.S. (2005). Phylogenetic analysis of classical swine fever virus in Taiwan. Arch. Virol..

[86] Deng M.C., Huang C.C., Huang T.S., Chang C.Y., Lin Y.J., Chien M.S., Jong M.H. (2005). Phylogenetic analysis of classical swine fever virus isolated from Taiwan. Vet. Microbiol..

[87] Lin Y.J., Chien M.S., Deng M.C., Huang C.C. (2007). Complete sequence of a subgroup 3.4 strain of classical swine fever virus from Taiwan. Virus Genes.

[88] Blacksell S.D., Khounsy S., Boyle D.B., Gleeson L.J., Westbury H.A., Mackenzie J.S. (2005). Genetic typing of classical swine fever viruses from Lao PDR by analysis of the 5' non-coding region. Virus Genes.

[89] Blacksell S.D., Khounsy S., Boyle D.B., Greiser-Wilke I., Gleeson L.J., Westbury H.A., Mackenzie J.S. (2004). Phylogenetic analysis of the *E2* gene of classical swine fever viruses from Lao PDR. Virus Res..

[90] Sakoda Y., Ozawa S., Damrongwatanapokin S., Sato M., Ishikawa K., Fukusho A. (1999). Genetic heterogeneity of porcine and ruminant pestiviruses mainly isolated in Japan. Vet. Microbiol..

[91] Coronado L., Liniger M., Munoz-Gonzalez S., Postel A., Perez L.J., Perez-Simo M., Perera C.L., Frias-Lepoureau M.T., Rosell R., Grundhoff A. (2017). Novel poly-uridine insertion in the 3' UTR and E2 amino acid substitutions in a low virulent classical swine fever virus. Vet. Microbiol..

[92] Lange M., Kramer-Schadt S., Blome S., Beer M., Thulke H.H. (2012). Disease severity declines over time after a wild boar population has been affected by classical swine fever--legend or actual epidemiological process?. Prev. Vet. Med..

[93] Dreier S., Zimmermann B., Moennig V., Greiser-Wilke I. (2007). A sequence database allowing automated genotyping of classical swine fever virus isolates. J. Virol. Methods.

[94] Greiser-Wilke I., Zimmermann B., Fritzemeier J., Floegel G., Moennig V. (2000). Structure and presentation of a world wide web database of CSF virus isolates held at the EU reference laboratory. Vet. Microbiol..

[95] Postel A., Schmeiser S., Zimmermann B., Becher P. (2016). The European classical swine fever virus database: Blueprint for a pathogen-specific sequence database with integrated sequence analysis tools. Viruses.

[96] Mayer D., Hofmann M.A., Tratschin J.D. (2004). Attenuation of classical swine fever virus by deletion of the viral N^pro^ gene. Vaccine.

[97] Risatti G.R., Borca M.V., Kutish G.F., Lu Z., Holinka L.G., French R.A., Tulman E.R., Rock D.L. (2005). The E2 glycoprotein of classical swine fever virus is a virulence determinant in swine. J. Virol..

[98] Tews B.A., Schurmann E.M., Meyers G. (2009). Mutation of cysteine 171 of pestivirus E^rns^ RNase prevents homodimer formation and leads to attenuation of classical swine fever virus. J. Virol..

[99] Meyers G., Saalmüller A., Büttner M. (1999). Mutations abrogating the RNAse activity in glycoprotein E^rns^ of the pestivirus classical swine fever virus lead to virus attenuation. J. Virol..

[100] Tamura T., Sakoda Y., Yoshino F., Nomura T., Yamamoto N., Sato Y., Okamatsu M., Ruggli N., Kida H. (2012). Selection of classical swine fever virus with enhanced pathogenicity reveals synergistic virulence determinants in E2 and NS4B. J. Virol..

[101] Risatti G.R., Holinka L.G., Fernandez Sainz I., Carrillo C., Kutish G.F., Lu Z., Zhu J., Rock D.L., Borca M.V. (2007). Mutations in the carboxyl terminal region of E2 glycoprotein of classical swine fever virus are responsible for viral attenuation in swine. Virology.

[102] Risatti G.R., Holinka L.G., Fernandez Sainz I., Carrillo C., Lu Z., Borca M.V. (2007). *N*-linked glycosylation status of classical swine fever virus strain brescia E2 glycoprotein influences virulence in swine. J. Virol..

[103] Sainz I.F., Holinka L.G., Lu Z., Risatti G.R., Borca M.V. (2008). Removal of a *N*-linked glycosylation site of classical swine fever virus strain Brescia E^rns^ glycoprotein affects virulence in swine. Virology.

[104] Tang F., Pan Z., Zhang C. (2008). The selection pressure analysis of classical swine fever virus envelope protein genes E^rns^ and E2. Virus Res..

[105] Wu Z., Wang Q., Feng Q., Liu Y., Teng J., Yu A.C., Chen J. (2010). Correlation of the virulence of CSFV with evolutionary patterns of E2 glycoprotein. Front. Biosci. (Elite Ed.).

[106] Ishikawa K., Nagai H., Katayama K., Tsutsui M., Tanabayashi K., Takeuchi K., Hishiyama M., Saitoh A., Takagi M., Gotoh K. (1995). Comparison of the entire nucleotide and deduced amino acid sequences of the attenuated hog cholera vaccine strain GPE- and the wild-type parental strain ALD. Arch. Virol..

[107] Jenckel M., Blome S., Beer M., Höper D. (2017). Quasispecies composition and diversity do not reveal any predictors for chronic classical swine fever virus infection. Arch. Virol..

[108] Petrov A., Blohm U., Beer M., Pietschmann J., Blome S. (2014). Comparative analyses of host responses upon infection with moderately virulent classical swine fever virus in domestic pigs and wild boar. Virol. J..

[109] Bunzenthal C. (2003). Determination of the virulence of classical swine fever virus isolates [Bestimmung der Virulenz von Virusisolaten der Klassischen Schweinepest]. Ph.D. Thesis.

[110] Floegel-Niesmann G., Blome S., Gerss-Dülmer H., Bunzenthal C., Moennig V. (2009). Virulence of classical swine fever virus isolates from Europe and other areas during 1996 until 2007. Vet. Microbiol..

[111] Floegel-Niesmann G., Bunzenthal C., Fischer S., Moennig V. (2003). Virulence of recent and former classical swine fever virus isolates evaluated by their clinical and pathological signs. J. Vet. Med. B Infect. Dis. Vet. Public Health.

[112] Tarradas J., de la Torre M.E., Rosell R., Perez L.J., Pujols J., Munoz M., Munoz I., Munoz S., Abad X., Domingo M. (2014). The impact of CSFV on the immune response to control infection. Virus Res..

[113] Depner K.R., Müller A., Gruber A., Rodriguez A., Bickhardt K., Liess B. (1995). Classical swine fever in wild boar (*Sus scrofa*)—Experimental infections and viral persistence. Dtsch. Tierarztl. Wochenschr..

[114] Kaden V., Steyer H., Schnabel J., Bruer W. (2005). Classical swine fever (CSF) in wild boar: The role of the transplacental infection in the perpetuation of CSF. J. Vet. Med. B Infect. Dis. Vet. Public Health.

[115] Kern B., Depner K.R., Letz W., Rott M., Thalheim S., Nitschke B., Plagemann R., Liess B. (1999). Incidence of classical swine fever (CSF) in wild boar in a densely populated area indicating CSF virus persistence as a mechanism for virus perpetuation. Zentralbl. Veterinarmed. B.

[116] von Benten K., Trautwein G., Richter-Reichhelm H.B., Liess B., Frey H.R. (1980). Experimental transplacental transmission of hog cholera virus in pigs. III. Histopathological findings in the fetus. Zentralbl. Veterinarmed. B.

[117] Stewart W.C., Carbrey E.A., Kresse J.I. (1973). Transplacental hog cholera infection in susceptible sows. Am. J. Vet. Res..

[118] Stewart W.C., Carbrey E.A., Kresse J.I. (1972). Transplacental hog cholera infection in immune sows. Am. J. Vet. Res..

[119] Richter-Reichhelm H.B., Trautwein G., von Benten K., Liess B., Frey H.R. (1980). Experimental transplacental transmission of hog cholera virus in pigs. II. Immunopathological findings in the fetus. Zentralbl. Veterinarmed. B.

[120] Overby E., Eskildsen M. (1977). Transplacental Infection in Susceptible Gilts after Inoculation with: I. Lapinized Swine Fever Vaccine, II. Bovine Viral Diarrhoea Virus Strains.

[121] Meyer H., Liess B., Frey H.R., Hermanns W., Trautwein G. (1981). Experimental transplacental transmission of hog cholera virus in pigs. IV. Virological and serological studies in newborn piglets. Zentralbl. Veterinarmed. B.

[122] Hermanns W., Trautwein G., Meyer H., Liess B. (1981). Experimental transplacental transmission of hog cholera virus in pigs. V. Immunopathological findings in newborn pigs. Zentralbl. Veterinarmed. B.

[123] Frey H.R., Liess B., Richter-Reichhelm H.B., von Benten K., Trautwein G. (1980). Experimental transplacental transmission of hog cholera virus in pigs. I. Virological and serological studies. Zentralbl. Veterinarmed. B.

[124] Cabezon O., Colom-Cadena A., Munoz-Gonzalez S., Perez-Simo M., Bohorquez J.A., Rosell R., Marco I., Domingo M., Lavin S., Ganges L. (2017). Post-natal persistent infection with classical swine fever virus in wild boar: A strategy for viral maintenance?. Transbound. Emerg. Dis..

[125] Munoz-Gonzalez S., Perez-Simo M., Munoz M., Bohorquez J.A., Rosell R., Summerfield A., Domingo M., Ruggli N., Ganges L. (2015). Efficacy of a live attenuated vaccine in classical swine fever virus postnatally persistently infected pigs. Vet. Res..

[126] Gomez-Villamandos J.C., Garcia de Leaniz I., Nunez A., Salguero F.J., Ruiz-Villamor E., Romero-Trevejo J.L., Sanchez-Cordon P.J. (2006). Neuropathologic study of experimental classical swine fever. Vet. Pathol..

[127] Van Oirschot J.T., Straw B.E., D’Allaire S., Mengeling W.L., Taylor D.J. (1999). Classial Swine Fever (Hog Cholera).

[128] Depner K.R., Lange E., Pontrakulpipat S., Fichtner D. (1999). Does porcine reproductive and respiratory syndrome virus potentiate classical swine fever virus infection in weaner pigs?. Zentralbl. Veterinarmed. B.

[129] Kaden V., Ziegler U., Lange E., Dedek J. (2000). Classical swine fever virus: Clinical, virological, serological and hematological findings after infection of domestic pigs and wild boars with the field isolate "Spante" originating from wild boar. Berl. Munch. Tierarztl. Wochenschr..

[130] Gomez-Villamandos J.C., Carrasco L., Bautista M.J., Sierra M.A., Quezada M., Hervas J., Chacon Mde L., Ruiz-Villamor E., Salguero F.J., Sonchez-Cordon P.J. (2003). African swine fever and classical swine fever: A review of the pathogenesis. Dtsch. Tierarztl. Wochenschr..

[131] Lange A., Blome S., Moennig V., Greiser-Wilke I. (2011). Pathogenesis of classical swine fever—Similarities to viral haemorrhagic fevers: A review. Berl. Munch. Tierarztl. Wochenschr..

[132] Liess B. (1987). Pathogenesis and epidemiology of hog cholera. Ann. Rech. Vet..

[133] Dunne H.W., Dunne H.W. (1970). Hog Cholera.

[134] Ressang A.A. (1973). Studies on the pathogenesis of hog cholera. II. Virus distribution in tissue and the morphology of the immune response. Zentralbl. Veterinarmed. B.

[135] Ressang A.A. (1973). Studies on the pathogenesis of hog cholera. I. Demonstration of hog cholera virus subsequent to oral exposure. Zentralbl. Veterinarmed. B.

[136] Bauhofer O., Summerfield A., McCullough K.C., Ruggli N. (2005). Role of double-stranded RNA and N^pro^ of classical swine fever virus in the activation of monocyte-derived dendritic cells. Virology.

[137] Carrasco C.P., Rigden R.C., Vincent I.E., Balmelli C., Ceppi M., Bauhofer O., Tache V., Hjertner B., McNeilly F., van Gennip H.G. (2004). Interaction of classical swine fever virus with dendritic cells. J. Gen. Virol..

[138] Fiebach A.R., Guzylack-Piriou L., Python S., Summerfield A., Ruggli N. (2011). Classical swine fever virus N^pro^ limits type I interferon induction in plasmacytoid dendritic cells by interacting with interferon regulatory factor 7. J. Virol..

[139] Jamin A., Gorin S., Cariolet R., Le Potier M.F., Kuntz-Simon G. (2008). Classical swine fever virus induces activation of plasmacytoid and conventional dendritic cells in tonsil, blood, and spleen of infected pigs. Vet. Res..

[140] Summerfield A., Ruggli N. (2015). Immune responses against classical swine fever virus: Between ignorance and lunacy. Front. Vet. Sci..

[141] Summerfield A., Alves M., Ruggli N., de Bruin M.G., McCullough K.C. (2006). High IFN-α responses associated with depletion of lymphocytes and natural IFN-producing cells during classical swine fever. J. Interferon Cytokine Res..

[142] Renson P., Blanchard Y., Le Dimna M., Felix H., Cariolet R., Jestin A., Le Potier M.F. (2010). Acute induction of cell death-related IFN stimulated genes (ISG) differentiates highly from moderately virulent CSFV strains. Vet. Res..

[143] Pauly T., König M., Thiel H.J., Saalmüller A. (1998). Infection with classical swine fever virus: Effects on phenotype and immune responsiveness of porcine T lymphocytes. J. Gen. Virol..

[144] Summerfield A., Knoetig S.M., Tschudin R., McCullough K.C. (2000). Pathogenesis of granulocytopenia and bone marrow atrophy during classical swine fever involves apoptosis and necrosis of uninfected cells. Virology.

[145] Susa M., König M., Saalmüller A., Reddehase M.J., Thiel H.J. (1992). Pathogenesis of classical swine fever: B-lymphocyte deficiency caused by hog cholera virus. J. Virol..

[146] Trautwein G. (1988). Classical swine fever and related infections. Pathology and Pathogenesis of the Disease.

[147] Bautista M.J., Ruiz-Villamor E., Salguero F.J., Sanchez-Cordon P.J., Carrasco L., Gomez-Villamandos J.C. (2002). Early platelet aggregation as a cause of thrombocytopenia in classical swine fever. Vet. Pathol..

[148] Heene D., Hoffmann-Fezer G., Müller-Berghaus G., Hoffmann R., Weiss E., Lasch H.G. (1971). Coagulation disorders in acute hog cholera. Beitr. Pathol..

[149] Weiss E., Teredesai A., Hoffmann R., Hoffmann-Fezer G. (1973). Volume distribution and ultrastructure of platelets in acute hog cholera. Thromb. Diath. Haemorrh..

[150] Blome S., Meindl-Böhmer A., Nowak G., Moennig V. (2013). Disseminated intravascular coagulation does not play a major role in the pathogenesis of classical swine fever. Vet. Microbiol..

[151] Bensaude E., Turner J.L., Wakeley P.R., Sweetman D.A., Pardieu C., Drew T.W., Wileman T., Powell P.P. (2004). Classical swine fever virus induces proinflammatory cytokines and tissue factor expression and inhibits apoptosis and interferon synthesis during the establishment of long-term infection of porcine vascular endothelial cells. J. Gen. Virol..

[152] Gomez-Villamandos J.C., Ruiz-Villamor E., Bautista M.J., Quezada M., Sanchez C.P., Salguero F.J., Sierra M.A. (2000). Pathogenesis of classical swine fever: Renal haemorrhages and erythrodiapedesis. J. Comp. Pathol..

[153] Hoffmann R., Hoffmann-Fezer G., Kimeto B., Weiss E. (1971). Microthrombi as morphological evidence of consumption coagulopathy in acute hog cholera. Zentralbl. Veterinarmed. B.

[154] Hulst M.M., Westra D.F., Wensvoort G., Moormann R.J. (1993). Glycoprotein E1 of hog cholera virus expressed in insect cells protects swine from hog cholera. J. Virol..

[155] Rümenapf T., Stark R., Meyers G., Thiel H.J. (1991). Structural proteins of hog cholera virus expressed by vaccinia virus: Further characterization and induction of protective immunity. J. Virol..

[156] König M., Lengsfeld T., Pauly T., Stark R., Thiel H.J. (1995). Classical swine fever virus: Independent induction of protective immunity by two structural glycoproteins. J. Virol..

[157] Paton D.J., Ibata G., Edwards S., Wensvoort G. (1991). An ELISA detecting antibody to conserved pestivirus epitopes. J. Virol. Methods.

[158] Graham S.P., Everett H.E., Haines F.J., Johns H.L., Sosan O.A., Salguero F.J., Clifford D.J., Steinbach F., Drew T.W., Crooke H.R. (2012). Challenge of pigs with classical swine fever viruses after C-strain vaccination reveals remarkably rapid protection and insights into early immunity. PLoS ONE.

[159] Renson P., Le Dimna M., Keranflech A., Cariolet R., Koenen F., Le Potier M.F. (2013). CP7_E2alf oral vaccination confers partial protection against early classical swine fever virus challenge and interferes with pathogeny-related cytokine responses. Vet. Res..

[160] van Oirschot J.T. (2003). Vaccinology of classical swine fever: From lab to field. Vet. Microbiol..

[161] Graham S.P., Haines F.J., Johns H.L., Sosan O., La Rocca S.A., Lamp B., Rümenapf T., Everett H.E., Crooke H.R. (2012). Characterisation of vaccine-induced, broadly cross-reactive IFN-γ secreting T cell responses that correlate with rapid protection against classical swine fever virus. Vaccine.

[162] Tarradas J., Argilaguet J.M., Rosell R., Nofrarias M., Crisci E., Cordoba L., Perez-Martin E., Diaz I., Rodriguez F., Domingo M. (2010). Interferon-γ induction correlates with protection by DNA vaccine expressing E2 glycoprotein against classical swine fever virus infection in domestic pigs. Vet. Microbiol..

[163] Ganges L., Barrera M., Nunez J.I., Blanco I., Frias M.T., Rodriguez F., Sobrino F. (2005). A DNA vaccine expressing the E2 protein of classical swine fever virus elicits T cell responses that can prime for rapid antibody production and confer total protection upon viral challenge. Vaccine.

[164] Blacksell S.D., Khounsy S., Van Aken D., Gleeson L.J., Westbury H.A. (2006). Comparative susceptibility of indigenous and improved pig breeds to classical swine fever virus infection: Practical and epidemiological implications in a subsistence-based, developing country setting. Trop. Anim. Health Prod..

[165] Everett H., Crooke H., Gurrala R., Dwarka R., Kim J., Botha B., Lubisi A., Pardini A., Gers S., Vosloo W. (2011). Experimental infection of common warthogs (*Phacochoerus africanus*) and bushpigs (*Potamochoerus larvatus*) with classical swine fever virus. I: Susceptibility and transmission. Transbound. Emerg. Dis..

[166] Terpstra C. (1987). Epizootiology of swine fever. Vet. Q..

[167] Weesendorp E., Landman W.J., Stegeman A., Loeffen W.L. (2008). Detection and quantification of classical swine fever virus in air samples originating from infected pigs and experimentally produced aerosols. Vet. Microbiol..

[168] Weesendorp E., Stegeman A., Loeffen W.L. (2009). Quantification of classical swine fever virus in aerosols originating from pigs infected with strains of high, moderate or low virulence. Vet. Microbiol..

[169] Laevens H., Koenen F., Deluyker H., de Kruif A. (1999). Experimental infection of slaughter pigs with classical swine fever virus: Transmission of the virus, course of the disease and antibody response. Vet. Rec..

[170] de Smit A.J., Bouma A., Terpstra C., van Oirschot J.T. (1999). Transmission of classical swine fever virus by artificial insemination. Vet. Microbiol..

[171] Floegel G., Wehrend A., Depner K.R., Fritzemeier J., Waberski D., Moennig V. (2000). Detection of classical swine fever virus in semen of infected boars. Vet. Microbiol..

[172] Moennig V., Greiser-Wilke I., Mahy B.W.J., van Regenmortel M.H.V. (2008). Classical swine fever virus. Encyclopedia of Virology.

[173] Pasick J., Brown C., Torres A. (2008). Classical swine fever. Foreign Animal Diseases.

[174] Hoffmann B., Beer M., Reid S.M., Mertens P., Oura C.A., van Rijn P.A., Slomka M.J., Banks J., Brown I.H., Alexander D.J. (2009). A review of RT-PCR technologies used in veterinary virology and disease control: Sensitive and specific diagnosis of five livestock diseases notifiable to the World Organisation for Animal Health. Vet. Microbiol..

[175] Hoffmann B., Beer M., Schelp C., Schirrmeier H., Depner K. (2005). Validation of a real-time RT-PCR assay for sensitive and specific detection of classical swine fever. J. Virol. Methods.

[176] Hoffmann B., Blome S., Bonilauri P., Fernandez-Pinero J., Greiser-Wilke I., Haegeman A., Isaksson M., Koenen F., Leblanc N., Leifer I. (2011). Classical swine fever virus detection: Results of a real-time reverse transcription polymerase chain reaction ring trial conducted in the framework of the European network of excellence for epizootic disease diagnosis and control. J. Vet. Diagn. Investig..

[177] Le Dimna M., Vrancken R., Koenen F., Bougeard S., Mesplede A., Hutet E., Kuntz-Simon G., Le Potier M.F. (2008). Validation of two commercial real-time RT-PCR kits for rapid and specific diagnosis of classical swine fever virus. J. Virol. Methods.

[178] Le Potier M.F., Le Dimna M., Kuntz-Simon G., Bougeard S., Mesplede A. (2006). Validation of a real-time RT-PCR assay for rapid and specific diagnosis of classical swine fever virus. Dev. Biol. (Basel).

[179] Leifer I., Blome S., Beer M., Hoffmann B. (2011). Development of a highly sensitive real-time RT-PCR protocol for the detection of classical swine fever virus independent of the 5′ untranslated region. J. Virol. Methods.

[180] McGoldrick A., Lowings J.P., Ibata G., Sands J.J., Belak S., Paton D.J. (1998). A novel approach to the detection of classical swine fever virus by RT-PCR with a fluorogenic probe (TaqMan). J. Virol. Methods.

[181] Paton D.J., McGoldrick A., Belak S., Mittelholzer C., Koenen F., Vanderhallen H., Biagetti M., De Mia G.M., Stadejek T., Hofmann M.A. (2000). Classical swine fever virus: A ring test to evaluate RT-PCR detection methods. Vet. Microbiol..

[182] Paton D.J., McGoldrick A., Bensaude E., Belak S., Mittelholzer C., Koenen F., Vanderhallen H., Greiser-Wilke I., Scheibner H., Stadejek T. (2000). Classical swine fever virus: A second ring test to evaluate RT-PCR detection methods. Vet. Microbiol..

[183] Belak S. (2007). Experiences of an oie collaborating centre in molecular diagnosis of transboundary animal diseases: A review. Dev. Biol. (Basel).

[184] Liu L., Luo Y., Accensi F., Ganges L., Rodriguez F., Shan H., Stahl K., Qiu H.J., Belak S. (2016). Pre-clinical evaluation of a real-time PCR assay on a portable instrument as a possible field diagnostic tool: Experiences from the testing of clinical samples for African and classical swine fever viruses. Transbound. Emerg. Dis..

[185] Chen H.T., Zhang J., Ma L.N., Ma Y.P., Ding Y.Z., Liu X.T., Chen L., Ma L.Q., Zhang Y.G., Liu Y.S. (2009). Rapid pre-clinical detection of classical swine fever by reverse transcription loop-mediated isothermal amplification. Mol. Cell. Probes.

[186] Chen L., Fan X.Z., Wang Q., Xu L., Zhao Q.Z., Zhou Y.C., Liu J., Tang B., Zou X.Q. (2010). A novel RT-LAMP assay for rapid and simple detection of classical swine fever virus. Virol. Sin..

[187] Chowdry V.K., Luo Y., Widen F., Qiu H.J., Shan H., Belak S., Liu L. (2014). Development of a loop-mediated isothermal amplification assay combined with a lateral flow dipstick for rapid and simple detection of classical swine fever virus in the field. J. Virol. Methods.

[188] Yin S., Shang Y., Zhou G., Tian H., Liu Y., Cai X., Liu X. (2010). Development and evaluation of rapid detection of classical swine fever virus by reverse transcription loop-mediated isothermal amplification (RT-LAMP). J. Biotechnol..

[189] Zhang X.J., Han Q.Y., Sun Y., Belak S., Liu L., Qiu H.J. (2011). Development of a loop-mediated isothermal amplification for visual detection of the HCLV vaccine against classical swine fever in China. J. Virol. Methods.

[190] Zhang X.J., Sun Y., Liu L., Belak S., Qiu H.J. (2010). Validation of a loop-mediated isothermal amplification assay for visualised detection of wild-type classical swine fever virus. J. Virol. Methods.

[191] Liu L., Xia H., Belak S., Widen F. (2009). Development of a primer-probe energy transfer real-time PCR assay for improved detection of classical swine fever virus. J. Virol. Methods.

[192] Zhang X.J., Xia H., Everett H., Sosan O., Crooke H., Belak S., Widen F., Qiu H.J., Liu L. (2010). Evaluation of a primer-probe energy transfer real-time PCR assay for detection of classical swine fever virus. J. Virol. Methods.

[193] Lung O., Pasick J., Fisher M., Buchanan C., Erickson A., Ambagala A. (2016). Insulated isothermal reverse transcriptase PCR (iiRT-PCR) for rapid and sensitive detection of classical swine fever virus. Transbound. Emerg. Dis..

[194] Turner L.W., Brown L.N., Carbrey E.A., Mengeling W.L., Perella D.H., Solorzano R.F. (1968). Recommended minimum standards for the isolation and identification of hog cholera by the fluorescent antibody-cell culture technique. Proc. Annu. Meet. U. S. Anim. Health Assoc..

[195] De Smit A.J., Eble P.L., de Kluijver E.P., Bloemraad M., Bouma A. (2000). Laboratory experience during the classical swine fever virus epizootic in the Netherlands in 1997–1998. Vet. Microbiol..

[196] Blome S., Meindl-Böhmer A., Loeffen W., Thuer B., Moennig V. (2006). Assessment of classical swine fever diagnostics and vaccine performance. Rev. Sci. Tech..

[197] Greiser-Wilke I., Blome S., Moennig V. (2007). Diagnostic methods for detection of classical swine fever virus--status quo and new developments. Vaccine.

[198] Floegel-Niesmann G. (2003). Marker vaccines and companion diagnostic tests for classical swine fever. Dev. Biol. (Basel).

[199] Floegel-Niesmann G. (2001). Classical swine fever (CSF) marker vaccine. Trial III. Evaluation of discriminatory ELISAs. Vet. Microbiol..

[200] Meyer D., Fritsche S., Luo Y., Engemann C., Blome S., Beyerbach M., Chang C.Y., Qiu H.J., Becher P., Postel A. (2017). The double-antigen ELISA concept for early detection of E^rns^ -specific classical swine fever virus antibodies and application as an accompanying test for differentiation of infected from marker vaccinated animals. Transbound. Emerg. Dis..

[201] Aebischer A., Müller M., Hofmann M.A. (2013). Two newly developed E^rns^-based ELISAs allow the differentiation of classical swine fever virus-infected from marker-vaccinated animals and the discrimination of pestivirus antibodies. Vet. Microbiol..

[202] Xia H., Harimoorthy R., Vijayaraghavan B., Blome S., Widen F., Beer M., Belak S., Liu L. (2015). Differentiation of classical swine fever virus infection from CP7_E2alf marker vaccination by a multiplex microsphere immunoassay. Clin. Vaccine Immunol..

[203] Huang Y.L., Pang V.F., Pan C.H., Chen T.H., Jong M.H., Huang T.S., Jeng C.R. (2009). Development of a reverse transcription multiplex real-time PCR for the detection and genotyping of classical swine fever virus. J. Virol. Methods.

[204] Leifer I., Depner K., Blome S., Le Potier M.F., Le Dimna M., Beer M., Hoffmann B. (2009). Differentiation of C-strain "Riems" or CP7_E2alf vaccinated animals from animals infected by classical swine fever virus field strains using real-time RT-PCR. J. Virol. Methods.

[205] Li Y., Zhao J.J., Li N., Shi Z., Cheng D., Zhu Q.H., Tu C., Tong G.Z., Qiu H.J. (2007). A multiplex nested RT-PCR for the detection and differentiation of wild-type viruses from C-strain vaccine of classical swine fever virus. J. Virol. Methods.

[206] Liu L., Hoffmann B., Baule C., Beer M., Belak S., Widen F. (2009). Two real-time RT-PCR assays of classical swine fever virus, developed for the genetic differentiation of naturally infected from vaccinated wild boars. J. Virol. Methods.

[207] Widen F., Everett H., Blome S., Fernandez Pinero J., Uttenthal A., Cortey M., von Rosen T., Tignon M., Liu L. (2014). Comparison of two real-time RT-PCR assays for differentiation of C-strain vaccinated from classical swine fever infected pigs and wild boars. Res. Vet. Sci..

[208] Zhao J.J., Cheng D., Li N., Sun Y., Shi Z., Zhu Q.H., Tu C., Tong G.Z., Qiu H.J. (2008). Evaluation of a multiplex real-time RT-PCR for quantitative and differential detection of wild-type viruses and C-strain vaccine of classical swine fever virus. Vet. Microbiol..

[209] Dietze K., Tucakov A., Engel T., Wirtz S., Depner K., Globig A., Kammerer R., Mouchantat S. (2017). Rope-based oral fluid sampling for early detection of classical swine fever in domestic pigs at group level. BMC Vet. Res..

[210] Michaud V., Gil P., Kwiatek O., Prome S., Dixon L., Romero L., Le Potier M.F., Arias M., Couacy-Hymann E., Roger F. (2007). Long-term storage at tropical temperature of dried-blood filter papers for detection and genotyping of RNA and DNA viruses by direct PCR. J. Virol. Methods.

[211] Mouchantat S., Globig A., Böhle W., Petrov A., Strebelow H.G., Mettenleiter T.C., Depner K. (2014). Novel rope-based sampling of classical swine fever shedding in a group of wild boar showing low contagiosity upon experimental infection with a classical swine fever field strain of genotype 2.3. Vet. Microbiol..

[212] Prickett J.R., Zimmerman J.J. (2010). The development of oral fluid-based diagnostics and applications in veterinary medicine. Anim. Health Res. Rev..

[213] Greiser-Wilke I., Moennig V. (2004). Vaccination against classical swine fever virus: Limitations and new strategies. Anim. Health Res. Rev..

[214] World Animal Health Information Database (WAHIS) Interface. http://www.oie.int/wahis_2/public/wahid.php/Diseasecontrol/measures.

[215] Kaden V., Lange E., Fischer U., Strebelow G. (2000). Oral immunisation of wild boar against classical swine fever: Evaluation of the first field study in Germany. Vet. Microbiol..

[216] Kaden V., Lange E., Küster H., Müller T., Lange B. (2010). An update on safety studies on the attenuated "RIEMSER Schweinepestoralvakzine" for vaccination of wild boar against classical swine fever. Vet. Microbiol..

[217] Milicevic V., Dietze K., Plavsic B., Tikvicki M., Pinto J., Depner K. (2012). Oral vaccination of backyard pigs against classical swine fever. Vet. Microbiol..

[218] Dietze K., Milicevic V., Depner K. (2013). Prospects of improved classical swine fever control in backyard pigs through oral vaccination. Berl. Munch. Tierarztl. Wochenschr..

[219] Monger V.R., Stegeman J.A., Dukpa K., Gurung R.B., Loeffen W.L. (2016). Evaluation of oral bait vaccine efficacy against classical swine fever in village backyard pig farms in Bhutan. Transbound. Emerg. Dis..

[220] Dahle J., Liess B. (1995). Assessment of safety and protective value of a cell culture modified strain "C" vaccine of hog cholera/classical swine fever virus. Berl. Munch. Tierarztl. Wochenschr..

[221] Kaden V., Riebe B. (2001). Classical swine fever (CSF): A historical review of research and vaccine production on the Isle of Riems. Berl. Munch. Tierarztl. Wochenschr..

[222] Terpstra C., Woortmeyer R., Barteling S.J. (1990). Development and properties of a cell culture produced vaccine for hog cholera based on the Chinese strain. Dtsch. Tierarztl. Wochenschr..

[223] Ferrari M. (1992). A tissue culture vaccine with lapinized Chinese (LC) strain of hog cholera virus (HCV). Comp. Immunol. Microbiol. Infect. Dis..

[224] Blome S., Gabriel C., Beer M. (2013). Possibilities and limitations in veterinary vaccine development using the example of classical swine fever. Berl. Munch. Tierarztl. Wochenschr..

[225] Ahrens U., Kaden V., Drexler C., Visser N. (2000). Efficacy of the classical swine fever (CSF) marker vaccine Porcilis Pesti in pregnant sows. Vet. Microbiol..

[226] Bouma A., De Smit A.J., De Jong M.C., De Kluijver E.P., Moormann R.J. (2000). Determination of the onset of the herd-immunity induced by the E2 sub-unit vaccine against classical swine fever virus. Vaccine.

[227] Bouma A., de Smit A.J., de Kluijver E.P., Terpstra C., Moormann R.J. (1999). Efficacy and stability of a subunit vaccine based on glycoprotein E2 of classical swine fever virus. Vet. Microbiol..

[228] de Smit A.J., Bouma A., de Kluijver E.P., Terpstra C., Moormann R.J. (2001). Duration of the protection of an E2 subunit marker vaccine against classical swine fever after a single vaccination. Vet. Microbiol..

[229] de Smit A.J., Bouma A., de Kluijver E.P., Terpstra C., Moormann R.J. (2000). Prevention of transplacental transmission of moderate-virulent classical swine fever virus after single or double vaccination with an E2 subunit vaccine. Vet. Q..

[230] Dewulf J., Laevens H., Koenen F., Vanderhallen H., Mintiens K., Deluyker H., de Kruif A. (2000). An experimental infection with classical swine fever in E2 sub-unit marker-vaccine vaccinated and in non-vaccinated pigs. Vaccine.

[231] Klinkenberg D., Moormann R.J., de Smit A.J., Bouma A., de Jong M.C. (2002). Influence of maternal antibodies on efficacy of a subunit vaccine: Transmission of classical swine fever virus between pigs vaccinated at 2 weeks of age. Vaccine.

[232] Lipowski A., Drexler C., Pejsak Z. (2000). Safety and efficacy of a classical swine fever subunit vaccine in pregnant sows and their offspring. Vet. Microbiol..

[233] Moormann R.J., Bouma A., Kramps J.A., Terpstra C., De Smit H.J. (2000). Development of a classical swine fever subunit marker vaccine and companion diagnostic test. Vet. Microbiol..

[234] van Aarle P. (2003). Suitability of an E2 subunit vaccine of classical swine fever in combination with the E^rns^-marker-test for eradication through vaccination. Dev. Biol. (Basel).

[235] van Oirschot J.T. (1999). DIVA vaccines that reduce virus transmission. J. Biotechnol..

[236] van Oirschot J.T. (2003). Emergency vaccination against classical swine fever. Dev. Biol. (Basel).

[237] Depner K.R., Bouma A., Koenen F., Klinkenberg D., Lange E., de Smit H., Vanderhallen H. (2001). Classical swine fever (CSF) marker vaccine. Trial II. Challenge study in pregnant sows. Vet. Microbiol..

[238] Beer M., Reimann I., Hoffmann B., Depner K. (2007). Novel marker vaccines against classical swine fever. Vaccine.

[239] Blome S., Moss C., Reimann I., König P., Beer M. (2017). Classical swine fever vaccines-state-of-the-art. Vet. Microbiol..

[240] Blome S., Aebischer A., Lange E., Hofmann M., Leifer I., Loeffen W., Koenen F., Beer M. (2012). Comparative evaluation of live marker vaccine candidates "CP7_E2alf" and "flc11" along with C-strain "Riems" after oral vaccination. Vet. Microbiol..

[241] Blome S., Gabriel C., Schmeiser S., Meyer D., Meindl-Böhmer A., Koenen F., Beer M. (2014). Efficacy of marker vaccine candidate CP7_E2alf against challenge with classical swine fever virus isolates of different genotypes. Vet. Microbiol..

[242] Dräger C., Petrov A., Beer M., Teifke J.P., Blome S. (2015). Classical swine fever virus marker vaccine strain CP7_E2alf: Shedding and dissemination studies in boars. Vaccine.

[243] Dräger C., Schröder C., König P., Tegtmeyer B., Beer M., Blome S. (2016). Efficacy of Suvaxyn CSF marker (CP7_E2alf) in the presence of pre-existing pestiviral antibodies against bovine viral diarrhea virus type 1. Vaccine.

[244] Eble P.L., Geurts Y., Quak S., Moonen-Leusen H.W., Blome S., Hofmann M.A., Koenen F., Beer M., Loeffen W.L. (2012). Efficacy of chimeric pestivirus vaccine candidates against classical swine fever: Protection and DIVA characteristics. Vet. Microbiol..

[245] Eble P.L., Quak S., Geurts Y., Moonen-Leusen H.W., Loeffen W.L. (2014). Efficacy of csf vaccine CP7_E2alf in piglets with maternally derived antibodies. Vet. Microbiol..

[246] Feliziani F., Blome S., Petrini S., Giammarioli M., Iscaro C., Severi G., Convito L., Pietschmann J., Beer M., De Mia G.M. (2014). First assessment of classical swine fever marker vaccine candidate CP7_E2alf for oral immunization of wild boar under field conditions. Vaccine.

[247] Gabriel C., Blome S., Urniza A., Juanola S., Koenen F., Beer M. (2012). Towards licensing of CP7_E2alf as marker vaccine against classical swine fever-duration of immunity. Vaccine.

[248] Goller K.V., Dräger C., Höper D., Beer M., Blome S. (2015). Classical swine fever virus marker vaccine strain CP7_E2alf: Genetic stability in vitro and in vivo. Arch. Virol..

[249] König P., Blome S., Gabriel C., Reimann I., Beer M. (2011). Innocuousness and safety of classical swine fever marker vaccine candidate CP7_E2alf in non-target and target species. Vaccine.

[250] König P., Hoffmann B., Depner K.R., Reimann I., Teifke J.P., Beer M. (2007). Detection of classical swine fever vaccine virus in blood and tissue samples of pigs vaccinated either with a conventional C-strain vaccine or a modified live marker vaccine. Vet. Microbiol..

[251] König P., Lange E., Reimann I., Beer M. (2007). CP7_E2alf: A safe and efficient marker vaccine strain for oral immunisation of wild boar against classical swine fever virus (CSFV). Vaccine.

[252] Leifer I., Lange E., Reimann I., Blome S., Juanola S., Duran J.P., Beer M. (2009). Modified live marker vaccine candidate CP7_E2alf provides early onset of protection against lethal challenge infection with classical swine fever virus after both intramuscular and oral immunization. Vaccine.

[253] Levai R., Barna T., Fabian K., Blome S., Belak K., Balint A., Koenen F., Kulcsar G., Farsang A. (2015). Pre-registration efficacy study of a novel marker vaccine against classical swine fever on maternally derived antibody negative (MDA-) target animals. Biologicals.

[254] Rangelova D., Nielsen J., Strandbygaard B., Koenen F., Blome S., Uttenthal A. (2012). Efficacy of marker vaccine candidate CP7_E2alf in piglets with maternally derived C-strain antibodies. Vaccine.

[255] Reimann I., Depner K., Trapp S., Beer M. (2004). An avirulent chimeric *Pestivirus* with altered cell tropism protects pigs against lethal infection with classical swine fever virus. Virology.

[256] Renson P., Le Dimna M., Gabriel C., Levai R., Blome S., Kulcsar G., Koenen F., Le Potier M.F. (2014). Cytokine and immunoglobulin isotype profiles during CP7_E2alf vaccination against a challenge with the highly virulent Koslov strain of classical swine fever virus. Res. Vet. Sci..

[257] Farsang A., Levai R., Barna T., Fabian K., Blome S., Belak K., Balint A., Koenen F., Kulcsar G. (2017). Pre-registration efficacy study of a novel marker vaccine against classical swine fever on maternally derived antibody positive (MDA+) target animals. Biologicals.

